# Correction: A novel role of MNT as a negative regulator of REL and the NF-κB pathway

**DOI:** 10.1038/s41389-021-00346-7

**Published:** 2021-08-02

**Authors:** Judit Liaño-Pons, M. Carmen Lafita-Navarro, Lorena García-Gaipo, Carlota Colomer, Javier Rodríguez, Alex von Kriegsheim, Peter J. Hurlin, Fabiana Ourique, M. Dolores Delgado, Anna Bigas, Lluis Espinosa, Javier León

**Affiliations:** 1 Departmento de Biología Molecular, Instituto de Biomedicina y Biotecnología de Cantabria (IBBTEC), CSIC-Universidad de Cantabria, Santander, Spain; 2 Cancer Research Program, Institut Hospital del Mar d’Investigacions Mèdiques, CIBERONC, Hospital del Mar, Barcelona, Spain; 3 Systems Biology Ireland, University College Dublin, Dublin, Ireland; 4 Shriners Hospitals for Children Research Center, Department of Cell, Developmental and Cancer Biology and Department of Orthopaedics and Rehabilitation, Oregon Health and Science University, Portland, OR USA; 5 Present Address: Department of Microbiology, Tumor and Cell Biology (MTC), Biomedicum B7, Karolinska Institutet, Stockholm, Sweden; 6 Present Address: Department of Cell Biology UT Southwestern Medical Center, Dallas, TX USA; 7 Present Address: Edinburgh Cancer Research Center, Institute of Genetics and Molecular Medicine, University of Edinburgh, Edinburgh, UK; 8 Present Address: Dept. of Biochemistry, Universidade Federal de Santa Catarina (UFSC), Florianópolis, Brazil

**Keywords:** Cancer genetics, Molecular biology, Cell growth

Correction to: *Oncogenesis* 10.1038/s41389-020-00298-4, published online 08 January 2021

Unfortunately, the spelling of the author’s name was incorrect Lluis Espinosa.

The original article has been corrected.

