# Supplementary material for: A novel role of MNT as a negative regulator of REL and the NF-κB pathway
Source: Oncogenesis. 2021 Jan 8;10(1):5. doi: 10.1038/s41389-020-00298-4 (PMC7794610; doi:10.1038/s41389-020-00298-4)
Supplement: Supplementary file 1 — Supplementary tables and figures [file 41389_2020_298_MOESM1_ESM.pdf]

Liaño-Pons et al. Supplementary data

Supplemental Table S1. MNT interactions detected by mass spectrometry. The table shows the MNT interacting proteins found by mass spectrometry of three MNT-immunoprecipitations from URMT (MAX-deficient) and three from URMax34 cells treated 24 h with 100 μM Zn_2SO_4 (expressing MAX). The fold change corresponds to the relative amount of the protein in the anti-MNT immunoprecipitates versus the IgG immunoprecipitates. The proteomic results are represented as the mean of the LFQ intensity of the immunoprecipitated proteins with an anti-MNT antibody. The interactions shown have *P*-values < 0.05.

	**Gene name**	**Protein name**	**Fold change (anti-MNT vs. IgG)**	**T-test (*P*-value)**
**URMT**	AMPD2	AMP deaminase 2	919.9	0.015
	MNT	MAX-binding protein MNT	182.67	0.006
	CCDC6	Coiled-coil domain-containing protein 6	49	0.015
	REL	Proto-oncogene c-REL	43.57	0.004
	QSER1	Glutamine and serine-rich protein 1	13.73	0.004
	MAT2A	S-adenosylmethionine synthase isoform type-2; S-adenosylmethionine synthase	6.43	0.002
	ALB	Serum albumin	5.85	0.024
	TPP2	Tripeptidyl-peptidase 2	3.99	0.02
	KRT6B	Keratin. type II cytoskeletal 6B	3.62	0.023
	PRKRA	Interferon-inducible double stranded RNA-dependent protein kinase activator A	3.14	0.024
	PDE4DIP	Myomegalin	2.68	0.018
	PIP	Prolactin-inducible protein	2.65	0.022
**URMax34**	NISCH	Nischarin	88.7	0
	AMPD2	AMP deaminase 2	43.72	0
	REL	Proto-oncogene c-Rel	36.99	0
	TPP2	Tripeptidyl-peptidase 2	34.75	0.002
	CCDC6	Coiled-coil domain-containing protein 6	30.83	0
	MAX	Protein max	26.3	0
	MNT	Max-binding protein MNT	18.26	0
	KIF7	Kinesin-like protein KIF7	9.96	0
	ARPC5	Actin-related protein 2/3 complex subunit 5	8.4	0.044
	QSER1	Glutamine and serine-rich protein 1	7.8	0
	SMARCE1	SWI/SNF-related matrix-associated actin-dependent regulator of chromatin subfamily E member 1	7.2	0
	SMARCC2;SMARCC1	SWI/SNF complex subunit SMARCC2;SWI/SNF complex subunit SMARCC1	6.77	0
	PGK1	Phosphoglycerate kinase 1;Phosphoglycerate kinase	5.99	0.047
	CCT5	T-complex protein 1 subunit epsilon	5.9	0.012
	TUBB2A;TUBB2B	Tubulin beta-2A chain;Tubulin beta-2B chain	5.62	0.001
	GSN	Gelsolin	5.12	0

VDAC2	Voltage-dependent anion-selective channel protein 2	5.1	0.019
VDAC1	Voltage-dependent anion-selective channel protein 1	5.07	0.049
HNRNPUL2;hCG_2044799	Heterogeneous nuclear ribonucleoprotein U-like protein 2	4.38	0.001
HSP90AA1	Heat shock protein HSP 90-alpha	4.06	0.035
S100A9	Protein S100-A9	4.02	0
DMBT1	Deleted in malignant brain tumors 1 protein	3.78	0.005
SBSN		3.75	0.037
PPIA	Peptidyl-prolyl cis-trans isomerase A;Peptidyl-prolyl cis-trans	3.74	0.02
CPSF7	Cleavage and polyadenylation specificity factor subunit 7	3.49	0
PKM2	Pyruvate kinase isozymes M1/M2;Pyruvate kinase	3.47	0.021
DSC3	Desmocollin-3 3.41 0.007	3.41	0.007
FABP5	Fatty acid-binding protein. epidermal	3.31	0.013
LDHA;LDHAL6A	L-lactate dehydrogenase A chain;L-lactate dehydrogenase;Llactate dehydrogenase A-like 6A	3.27	0.007
HSP90AB1	Heat shock protein HSP 90-beta	3.2	0.033
SUPT16H	FACT complex subunit SPT16	3.12	0.041
RAB39A;RAB6A;RAB6B	Ras-related protein Rab-39A;Ras-related protein Rab-6A;Rasrelated protein Rab-6B	3.02	0.006
HSPB1	Heat shock protein beta-1	2.74	0
ENO1	Alpha-enolase	2.56	0.007
PRDX1	Peroxiredoxin-1	2.51	0.011
PPP1R12A	Protein phosphatase 1 regulatory subunit 12A	2.45	0.022
ATP5B	ATP synthase subunit beta. mitochondrial;ATP synthase subunit beta	2.42	0.018
HSPA8	Heat shock cognate 71 kDa protein	2.42	0.003
AZGP1	Zinc-alpha-2-glycoprotein	2.42	0.008
HSPA5	78 kDa glucose-regulated protein	2.29	0
ATP5A1	ATP synthase subunit alpha. mitochondrial;ATP synthase subunit alpha	2.25	0.008
ANXA2;ANXA2P2	Annexin A2;Annexin;Putative annexin A2-like protein	2.2	0.028
CCT8	T-complex protein 1 subunit theta	2.19	0.036
NUDT21;DKFZp313O211	Cleavage and polyadenylation specificity factor subunit 5	2.18	0
METAP1	Methionine aminopeptidase 1;Methionine aminopeptidase	2.15	0.039
CFL1	Cofilin-1	2.06	0.027
DSG1	Desmoglein-1	2.06	0.021
DSC1	Desmocollin-1	2.05	0.036

Supplemental Table S2. Primers used in this study. The genes, primer sequences and their application in this study are indicated. The primers target human genes and their melting temperature (Tm) is 60 ºC.. The genome coordinates (being +1 the transcription start site) are also indicated in the amplicons for ChIP. ChIP, chromatin immunoprecipitation; RT-qPCR, reverse transcription-quantitative polymerase chain reaction

Gene	**Primers Sequence (5' → 3')**	**Use**
BCL2L1	ACATCCCAGCTCCACATCAC<br>AAGAGTGAGCCCAGCAGAAC	RT-qPCR
CCL5	TACACCAGTGGCAAGTGCTC<br>GCAAGCAGAAACAGGCAAAT	RT-qPCR
IL6	AGTGAGGAACAAGCCAGAGC<br>GAGGTGCCCATGCTACATTT	RT-qPCR
IL8	TTTGCCAAGGAGTGCTAAAGA<br>ACTTCTCCACAACCCTCTGC	RT-qPCR
MNT	AGCCAGTGGATGGACGTACT<br>GACGATGGCTCAGCTTAGGT	RT-qPCR
NFKB1	CATCCCATGGTGGACTACCT<br>ACAGTGCAGATCCCATCCTC	RT-qPCR
NFKBIA	TGAAGAAAAGGCACTGACCA<br>CTCACAGGCAAGGTGTAGGG	RT-qPCR
REL	GAACGATTGGGAAGCAAAAG<br>GGCACAGTTTCTGGAAAAGC	RT-qPCR
RELA	GGCGAGAGGAGCACAGATAC<br>CAGCCTCATAGAAGCCATCC	RT-qPCR
RPS14	TCACCGCCCTACACATCAAACT<br>CTGCGAGTGCTGTCAGAGG	RT-qPCR
MNT -842/-619 bp	ATGTGACCTGCAGACACTGG<br>GCGACTGGAGACTGTCAAGA	ChIP
MNT -4729/-4549 bp	GAGTTCCGCTCTGTTTGCTT<br>GCTGCAGGATGAAGAGGAAA	ChIP
NFKBIA -743/-937 bp	GTGCCCAGAAGTAGGCTCAC<br>TGGGGAAACTGCTGAATAGG	ChIP
NFKBIA -275/-476 bp	CCAGCCATCATTTCCACTCT<br>CCTGCACCCTGTAATCCTGT	ChIP
NFKBIA -67/-316 bp	AGAAGGCTCACTTGCAGAGG<br>GGAATTTCCAAGCCAGTCAG	ChIP
NFKBIA +171/+343 bp	AGAAGGAGCGGCTACTGGAC<br>ACTTACGAGTCCCCGTCCTC	ChIP
NFKBIA +801/+1003 bp	GCCAGGAACACTCAGCTCAT<br>CCATGGTCAGTGCCTTTTCT	ChIP
NFKBIA +1959/+2176 bp	CTTGGGTGCTGATGTCAATG<br>CCCCACACTTCAACAGGAGT	ChIP
TXNIP -156/+51 bp	TCCAGAGCGCAACAACCAT<br>AAGCAGGAGGCGGAAACGT	ChIP

Supplemental Table S3. Primary and secondary antibodies used in this study. aa, amino acids; IB, immunoblot; IF, immunofluorescence; IP, immunoprecipitation; PLA, proximity ligation assay; ChIP, chromatin immunoprecipitation; RRID, Research Resource Identifier.

**Primary antibodies**					
**Antigen**	**Immunogen**	**Type**	**Origin (reference)**	**RRID**	**Technique and dilution**
β-actin	357-375 aa (human)	Mouse monoclonal	Santa Cruz Biotech. (C-2, sc-8432)	AB_6266 30	IB (1:3000)
HA	Influenza virus hemagglutinin (HA) epitope	Mouse Monoclonal	Genecopiea (CGAB-HA-0050)	AB_2801 472	IB (1:1000); IP
HA	Influenza virus hemagglutinin (HA) epitope	Rat monoclonal	Roche (3F10)	AB_2314 622	IB (1:2000)
IκBα	C-terminus (human)	Rabbit polyclonal	Cell Signaling (#4812)	AB_1069 4416	IB (1:1000)
IgG	-	Rabbit polyclonal	Cell Signaling (#2729)	AB_1031 062	IP; ChIP
IgG	-	Mouse polyclonal	Santa Cruz Biotech. (sc-2025)	AB_7371 82	IP; ChIP
MAX	C-terminal (human)	Rabbit polyclonal	Santa Cruz Biotech. (C-17, sc-197)	AB_2281 783	IB (1:1000); IP, PLA (1:200)
MNT	226-361 aa (human)	Rabbit polyclonal	Santa Cruz Biotech. (M-132, sc-769)	AB_2145 079	IB (1:1000); IF (1:200); IP, PLA (1:200)
MNT	1-50 aa (human)	Rabbit polyclonal	Novus (NBP2-04052)	AB_2810 951	IB (1:1000); IP; ChIP
MNT	532-582 aa (human)	Rabbit polyclonal	Novus (NBP2-04053)	AB_2801 473	IB (1:1000); IP; ChIP
MYC	Full-length (human)	Mouse monoclonal	Santa Cruz Biotech. (C33, sc-42)	AB_2282 408	PLA (1:100)
p65	C-terminus (human)	Goat polyclonal	Santa Cruz Biotech. (C-20, sc-372-G)	AB_6320 37	IB (1:1000), IF (1:200), IP
p65	N-terminus	Rabbit polyclonal	Santa Cruz Biotech. (A, sc-109)	AB_6320 39	PLA (1:200)

p105/p50	N-terminus (human)	Rabbit polyclonal	Cell Signaling (#3035)	AB_330564	IB (1:1000); IP
REL	N-terminus (human)	Rabbit polyclonal	Santa Cruz Biotech. (N, sc-70)	AB_2178727	IB (1:1000) IF (1:200), IP, PLA (1:200), ChIP
REL	143-184 aa (human)	Mouse monoclonal	Santa Cruz Biotech. (D-6, sc-373713)	AB_10919139	IB (1:500), PLA (1:100)
REL	C-terminus (human)	Rabbit polyclonal	Cell Signaling (#4727)	AB_2178843	IB (1:1000); IP
RhoGDI	N-terminal (human)	Rabbit polyclonal	Santa Cruz Biotech (A-20, sc-360)	AB_2227516	IB (1:1000)
SIN3B	172-228 aa (mouse)	Mouse monoclonal	Santa Cruz Biotech (H-4, sc-13145)	AB_628254	IB (1:1000)
**Secondary antibodies**					
**Antibody**	**Immunogen**	**Type**	**Origin (reference)**	**RRID**	**Technique and dilution**
Anti-Goat IRDye®680	goat heavy and light immunoglobulins chains	Donkey polyclonal	Li-Cor Biosciences (926-68074)	AB_10956736	IB (1:10000)
Anti-Goat IRDye®800	goat heavy and light immunoglobulins chains	Donkey polyclonal	Li-Cor Biosciences (926-32214)	AB_621846	IB (1:10000)
Anti-Mouse IRDye®680	mouse heavy and light immunoglobulins chains	Donkey polyclonal	Li-Cor Biosciences (926-68072)	AB_10953628	IB (1:10000)
Anti-Mouse IRDye®800	mouse heavy and light immunoglobulins chains	Donkey polyclonal	Li-Cor Biosciences (926-32212)	AB_621847	IB (1:10000)
Anti-Rabbit IRDye®680	rabbit heavy and light immunoglobulins chains	Donkey polyclonal	Li-Cor Biosciences (926-68073)	AB_10954442	IB (1:10000)
Anti-Rabbit IRDye®800	rabbit heavy and light immunoglobulins chains	Donkey polyclonal	Li-Cor Biosciences (926-32213)	AB_621848	IB (1:10000)
Anti-Rabbit FITC	rabbit heavy and light immunoglobulins chains	Goat polyclonal	Jackson InmunoResearch laboratories (111-095-045)	AB_2337975	IF (1:200)

Supplementary figure S1

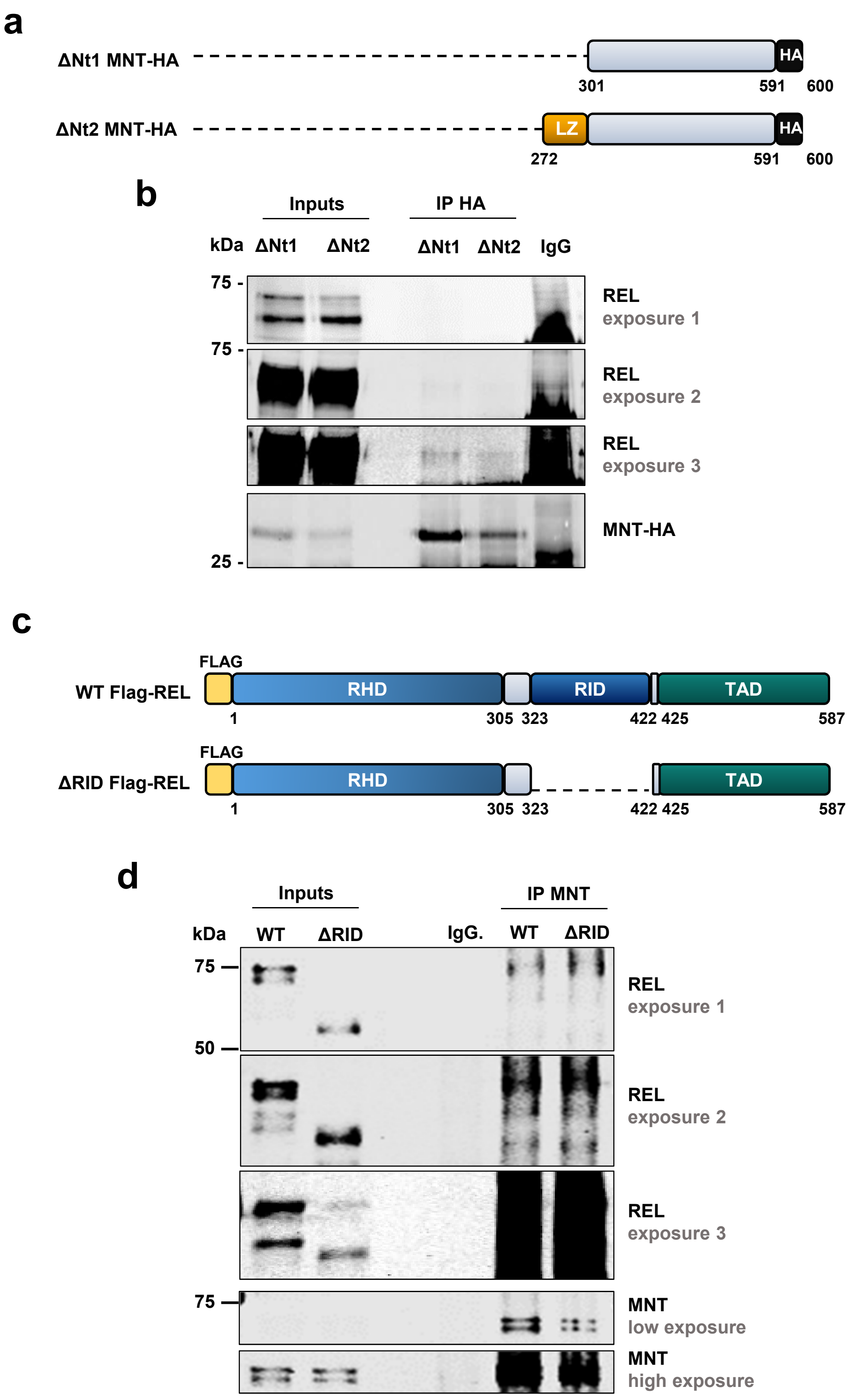

Supplementary figure S1. (a) Schematic representation of the mouse MNT deletion constructs used for the co-IP assays, ΔNt1 and ΔNt2 MNT-HA. **(b)** C6 cell lysates 48 h after transfection with REL-flag (mouse) and ΔNt1 or ΔNt2 MNT-HA (mouse) were immunoprecipitated with anti-HA antibodies (IgG as negative control). The immunoblots of HA and REL (three different exposures) are shown. **(c)** Schematic representation of the human REL constructs used for the co-IP assays, WT Flag-REL and ΔRID Flag-REL (amino acids 323-422). C6 cell lysates 48 h after transfection with WT MNT-HA and either WT Flag-REL or ΔRID Flag-REL were immunoprecipitated with anti-MNT antibodies (IgG as negative control). The immunoblots of REL (three exposures) and MNT (low and high exposure) are shown.

Supplementary figure S2

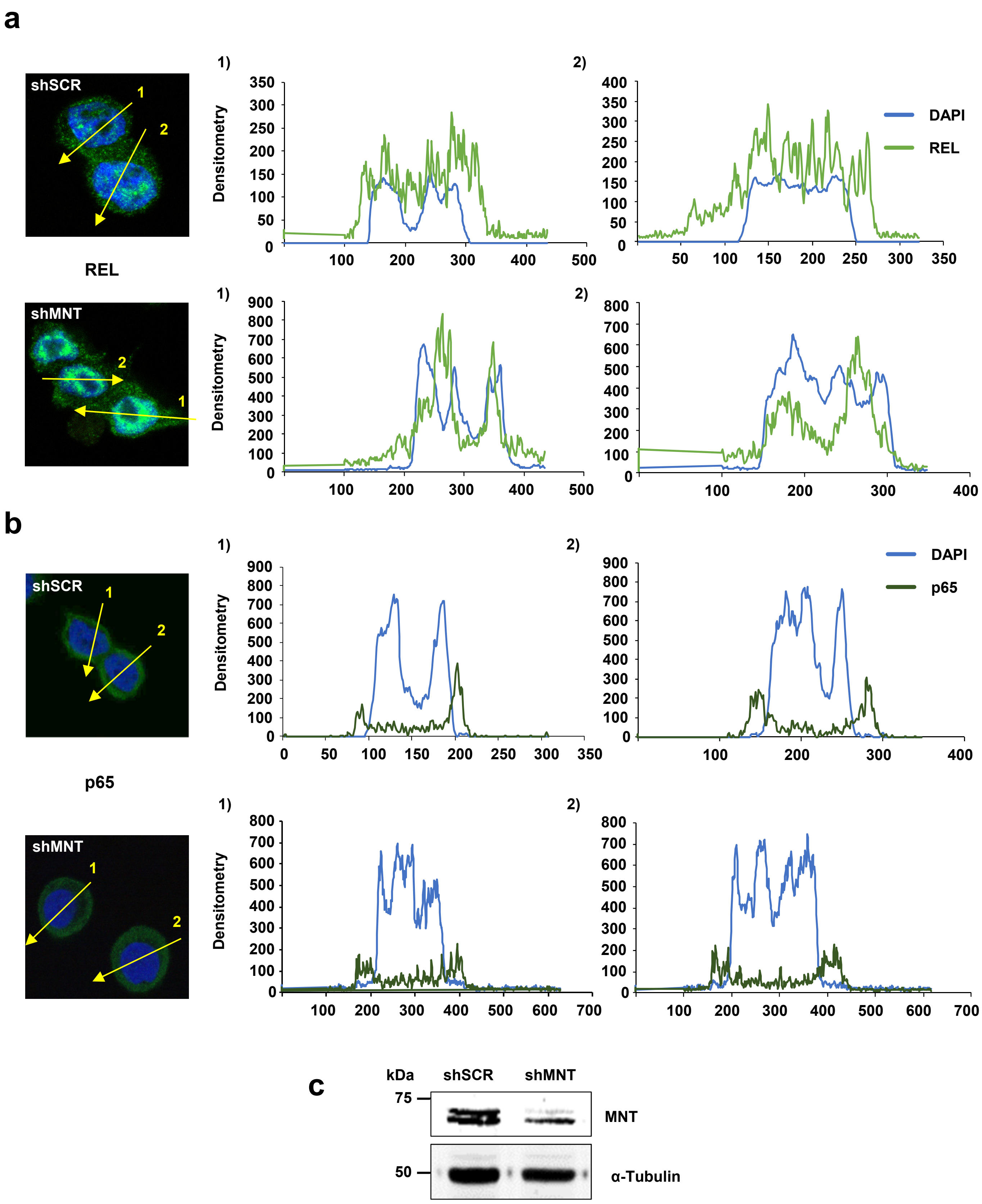

Supplementary figure S2. (a) REL immunofluorescence and **(b)** p65 immunofluorescence in LoVo cells that were infected with lentiviral particles carrying two shRNAs against MNT (shMNT) or a scrambled control shRNA (shSCR) and selected with puromycin (1 µg/mL) for 72 h. The signal was quantified with the ImageJ software. **(c)** Immunoblot of the cells used for the immunofluorescence showing the *MNT* knockdown efficiency. α-Tubulin was determined as a protein loading control.

Supplementary figure S3

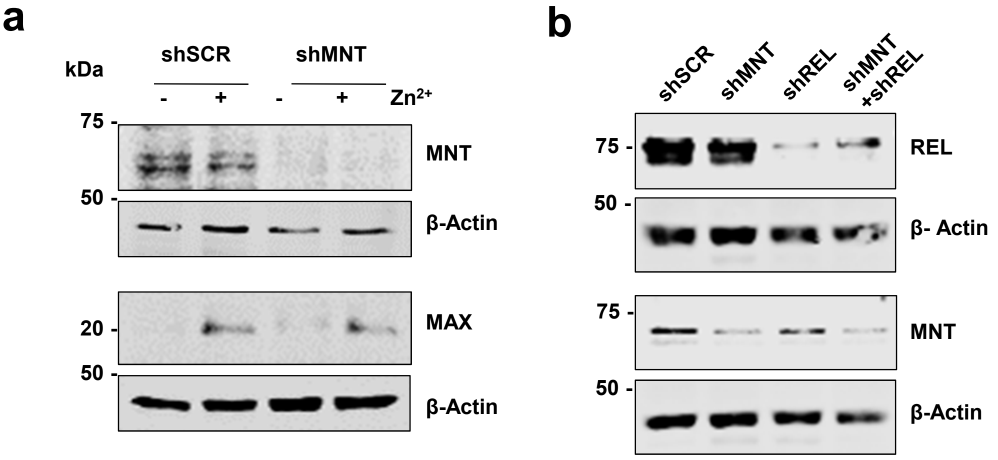

Supplementary figure S3. (a) Immunoblot of the URMax34 cells used for the luciferase assay in Fig. 4d showing MNT and MAX expression. URMax34 cells were transfected with the shRNAs and luciferase constructs and harvested 72 h after the transfection and 24 h of 100 μM $ZnSO_4$ treatment. β-Actin was determined as a protein loading control for each different gel. **(b)** Immunoblot of LoVo cells used for the colony assays in Fig. 4e. LoVo cells were infected with lentivirus expressing short hairpin (sh) RNA sequences for silencing MNT and REL as indicated. Cell were harvested 72 h after infection and the protein levels of REL, MNT and β-Actin (loading control) were determined.
