# Supplementary material for: A novel role of MNT as a negative regulator of REL and the NF-κB pathway
Source: Oncogenesis. 2021 Jan 8;10(1):5. doi: 10.1038/s41389-020-00298-4 (PMC7794610; doi:10.1038/s41389-020-00298-4)
Supplement: Supplementary file 2 — Authors agreements with changes [file 41389_2020_298_MOESM2_ESM.pdf]

Leon Serrano, Javier

De: Judit Liano Pons <judit.liano.pons@ki.se>
Enviado el: jueves, 26 de noviembre de 2020 14:04
Para: Leon Serrano, Javier
Asunto: Re: ONCSIS-20-0232RR Initial Quality Check

Dear Javier,

I agree with the changes of the author list.

Best,
Judit Liano

Judit Liano Pons, PhD
Postdoctoral researcher | Marie Arsenian Henriksson's group

Karolinska Institutet | Dept. of Microbiology, Tumor and Cell Biology (MTC)
Biomedicum - Solnavägen 9, B7
171 65 Solna (Sweden)

Phone: 0046-0-737608211 | judit.liano.pons@ki.se

From: Leon Serrano, Javier <javier.leon@unican.es>
Date: Thursday, 26 November 2020 at 12:22
To: Judit Liano Pons <judit.liano.pons@ki.se>, MariadelCarmen.LafitaNavarro@UTSouthwestern.edu <MariadelCarmen.LafitaNavarro@UTSouthwestern.edu>, Garcia Gaipo, Lorena <lorena.garcia@unican.es>, carlotacolomer@gmail.com <carlotacolomer@gmail.com>, Rodriguez Martinez, Javier <javier.rodriguez@unican.es>, Alex.VonKriegsheim@igmm.ed.ac.uk <Alex.VonKriegsheim@igmm.ed.ac.uk>, phurlin@shrinenet.org <phurlin@shrinenet.org>, fabiana.ourique@posgrad.ufsc.br <fabiana.ourique@posgrad.ufsc.br>, Delgado Villar, Maria Dolores <maria.delgado@unican.es>, abigas@imim.es <abigas@imim.es>, lespinosa@imim.es <lespinosa@imim.es>, Leon Serrano, Javier <javier.leon@unican.es>
Subject: RV: ONCSIS-20-0232RR Initial Quality Check

Dear author
The journal Oncogenesis is asking for the agreement of all the authors to the author list of the resubmitted version of the manuscript
As you can see in their instructions below, I would need that you send me a simple email confirming that you agree with the changes. I will combine your response emails in a document and submit it to the journal. I attach the final version and I copy the list of authors below

Judit Liaño-Pons1,5, M. Carmen Lafita-Navarro1,6, Lorena García-Gaipo1, Carlota Colomer2, Javier Rodríguez3, Alex von Kriegsheim3, Peter Hurlin4, Fabiana Ourique1,7, M. Dolores Delgado1, Anna Bigas2, M. Lluis Espinosa2 and Javier Leon1

Best regards
Javier

Prof. Javier León
Instituto de Biomedicina y Biotecnología de Cantabria (IBBTEC)
C/ Albert Einstein 22, 39011 Santander

Universidad de Cantabria-CSIC-Sodercan
Tel 34 942 201952; Mov 34 637 858 317

prohibited. If you have received this email in error please notify our Manuscript Tracking System Helpdesk team at http://platformsupport.nature.com .

Details of the confidentiality and pre-publicity policy may be found here http://www.nature.com/authors/policies/confidentiality.html

Privacy Policy | Update Profile

När du skickar e-post till Karolinska Institutet (KI) innebär detta att KI kommer att behandla dina personuppgifter. Här finns information om hur KI behandlar personuppgifter.

Sending email to Karolinska Institutet (KI) will result in KI processing your personal data. You can read more about KI's processing of personal data here.

Leon Serrano, Javier

**De:**	Maria del Carmen Lafita Navarro <MariadelCarmen.LafitaNavarro@UTSouthwestern.edu>
**Enviado el:**	jueves, 26 de noviembre de 2020 16:17
**Para:**	Leon Serrano, Javier
**Asunto:**	Re: ONCSIS-20-0232RR Initial Quality Check

I agree with the new author list of the "A novel role of MNT as a negative regulator of REL and the NF-κB pathway" manuscript.

M.Carmen Lafita Navarro

On Nov 26, 2020, at 5:21 AM, Leon Serrano, Javier <javier.leon@unican.es> wrote:

EXTERNAL MAIL

Dear author
The journal Oncogenesis is asking for the agreement of all the authors to the author list of the resubmitted version of the manuscript
As you can see in their instructions below, I would need that you send me a simple email confirming that you agree with the changes. I will combine your response emails in a document and submit it to the journal. I attach the final version and I copy the list of authors below

Judit Liaño-Pons1,5, M. Carmen Lafita-Navarro1,6, Lorena García-Gaipo1, Carlota Colomer2, Javier Rodríguez3, Alex von Kriegsheim3, Peter Hurlin4, Fabiana Ourique1,7, M. Dolores Delgado1, Anna Bigas2, M. Lluis Espinosa2 and Javier Leon1

Best regards
Javier

<image003.jpg>
Prof. Javier León
Instituto de Biomedicina y Biotecnología de Cantabria (IBBTEC)
C/ Albert Einstein 22, 39011 Santander
Universidad de Cantabria-CSIC-Sodercan
Tel 34 942 201952; Mov 34 637 858 317

De: oncogenesis@nature.com <oncogenesis@nature.com>
Enviado el: jueves, 26 de noviembre de 2020 11:30
Para: Leon Serrano, Javier <javier.leon@unican.es>
Asunto: ONCSIS-20-0232RR Initial Quality Check

Dear Professor León,

In checking in your manuscript submitted to Oncogenesis it has come to our attention that the following must be addressed before we can begin the peer review process.

It has come to our attention that your most recent author list differs from the one in your original submission.

Please request agreement from all authors including additions and deletions, these can be collected in the following way:

Email your co-authors with the change, and ask them to reply to your email confirming that

Leon Serrano, Javier

De: Garcia Gaipo, Lorena
Enviado el: jueves, 26 de noviembre de 2020 18:13
Para: Leon Serrano, Javier
Asunto: RE: ONCSIS-20-0232RR Initial Quality Check
Datos adjuntos: image001.jpg

Dejar Professor León,

I confirm that I agree with the changes in the author list of the manuscript.

Lorna García-Gaipo

De: Leon Serrano, Javier <javier.leon@unican.es>
Enviado el: jueves, 26 de noviembre de 2020 12:21
Para: judit.liano.pons@ki.se; MariadelCarmen.LafitaNavarro@UTSouthwestern.edu; Garcia Gaipo, Lorena <lorena.garcia@unican.es>; carlotacolomer@gmail.com; Rodriguez Martinez, Javier <javier.rodriguez@unican.es>; Alex.VonKriegsheim@igmm.ed.ac.uk; phurlin@shrinenet.org; fabiana.ourique@posgrad.ufsc.br; Delgado Villar, Maria Dolores <maria.delgado@unican.es>; abigas@imim.es; lespinosa@imim.es; Leon Serrano, Javier <javier.leon@unican.es>
Asunto: RV: ONCSIS-20-0232RR Initial Quality Check

Dear author

The journal Oncogenesis is asking for the agreement of all the authors to the author list of the resubmitted version of the manuscript

As you can see in their instructions below, I would need that you send me a simple email confirming that you agree with the changes. I will combine your response emails in a document and submit it to the journal. I attach the final version and I copy the list of authors below

Judit Liaño-Pons1,5, M. Carmen Lafita-Navarro1,6, Lorena García-Gaipo1, Carlota Colomer2, Javier Rodríguez3, Alex von Kriegsheim3, Peter Hurlin4, Fabiana Ourique1,7, M. Dolores Delgado1, Anna Bigas2, M. Lluis Espinosa2 and Javier Leon1

Best regards

Javier

Leon Serrano, Javier

De: Carlota Colomer <carlotacolomer@gmail.com>
Enviado el: viernes, 27 de noviembre de 2020 11:51
Para: Leon Serrano, Javier
Asunto: Re: ONCSIS-20-0232RR Initial Quality Check

Dear Javier,

I agree with the changes in the author list for the manuscript "A novel role of MNT as a negative regulator of REL and the NF-κB pathway" by Liaño-Pons et al.

Thanks you & Best regards,

Carlota Colomer

PD: Felicidades!

El jue, 26 nov 2020 a las 12:21, Leon Serrano, Javier (<javier.leon@unican.es>) escribió:

> Dear author
>
> The journal Oncogenesis is asking for the agreement of all the authors to the author list of the resubmitted version of the manuscript
>
> As you can see in their instructions below, I would need that you send me a simple email confirming that you agree with the changes. I will combine your response emails in a document and submit it to the journal. I attach the final version and I copy the list of authors below
>
> Judit Liaño-Pons1,5, M. Carmen Lafita-Navarro1,6, Lorena García-Gaipo1, Carlota Colomer2, Javier Rodríguez3, Alex von Kriegsheim3, Peter Hurlin4, Fabiana Ourique1,7, M. Dolores Delgado1, Anna Bigas2, M. Lluis Espinosa2 and Javier Leon1
>
> Best regards
>
> Javier

Prof. Javier León
Instituto de Biomedicina y Biotecnología de Cantabria (IBBTEC)
C/ Albert Einstein 22, 39011 Santander

Universidad de Cantabria-CSIC-Sodercan

Tel 34 942 201952; Mov 34 637 858 317

Leon Serrano, Javier

**De:**	Rodriguez Martinez, Javier
**Enviado el:**	viernes, 27 de noviembre de 2020 15:37
**Para:**	Leon Serrano, Javier
**Asunto:**	Fwd: Agreeement on author list-RECORDATORIO

Javier Rodríguez agrees with the new author list of the resubmitted version of the manuscript ONCSIS-20-0232RR

Obtener [Outlook para iOS]

De: Rodriguez Martinez, Javier <javier.rodriguez@unican.es>
Enviado: viernes, noviembre 27, 2020 2:30 p. m.
Para: Leon Serrano, Javier
Asunto: Re: Agreeement on author list-RECORDATORIO

Me, Javier Rodríguez agree with the new version of the manuscript ONCSIS-20-0232RR

Obtener [Outlook para iOS]

De: Leon Serrano, Javier <javier.leon@unican.es>
Enviado: Friday, November 27, 2020 12:36:55 PM
Para: Rodriguez Martinez, Javier <javier.rodriguez@unican.es>
Asunto: RV: Agreeement on author list-RECORDATORIO

Dear author
The journal Oncogenesis is asking for the agreement of all the authors to the author list of the resubmitted version of the manuscript
As you can see in their instructions below, I would need that you send me a simple email confirming that you agree with the changes. I will combine your response emails in a document and submit it to the journal. I attach the final version and I copy the list of authors below

Judit Liaño-Pons1,5, M. Carmen Lafita-Navarro1,6, Lorena García-Gaipo1, Carlota Colomer2, Javier Rodríguez3, Alex von Kriegsheim3, Peter Hurlin4, Fabiana Ourique1,7, M. Dolores Delgado1, Anna Bigas2, M. Lluis Espinosa2 and Javier Leon1

Best regards
Javier

ibbtec

Prof. Javier León
Instituto de Biomedicina y Biotecnologia de Cantabria (IBBTEC)
C/ Albert Einstein 22, 39011 Santander
CSIC-UNIVERSIDAD DE CANTABRIA-SODERCAN

Tel. + 34 942 201952/ Mov. + 34 637 858 317
Email: leonj@unican.es

Leon Serrano, Javier

De: VON KRIEGSHEIM Alex <Alex.VonKriegsheim@igmm.ed.ac.uk>
Enviado el: jueves, 26 de noviembre de 2020 15:21
Para: Leon Serrano, Javier
Asunto: Re: ONCSIS-20-0232RR Initial Quality Check

Dear Javier,
all agreed
best
Alex

Alex von Kriegsheim
Edinburgh Cancer Research UK Centre
Institute of Genetics and Molecular Medicine
University of Edinburgh
Crewe Road South
Edinburgh
EH4 2XR
United Kingdom
tel: +44 131 651 8566

From: Leon Serrano, Javier <javier.leon@unican.es>
Sent: 26 November 2020 11:21
To: judit.liano.pons@ki.se <judit.liano.pons@ki.se>; MariadelCarmen.LafitaNavarro@UTSouthwestern.edu <MariadelCarmen.LafitaNavarro@UTSouthwestern.edu>; Garcia Gaipo, Lorena <lorena.garcia@unican.es>; carlotacolomer@gmail.com <carlotacolomer@gmail.com>; Rodriguez Martinez, Javier <javier.rodriguez@unican.es>; VON KRIEGSHEIM Alex <Alex.VonKriegsheim@igmm.ed.ac.uk>; phurlin@shrinenet.org <phurlin@shrinenet.org>; fabiana.ourique@posgrad.ufsc.br <fabiana.ourique@posgrad.ufsc.br>; Delgado Villar, Maria Dolores <maria.delgado@unican.es>; abigas@imim.es <abigas@imim.es>; lespinosa@imim.es <lespinosa@imim.es>; Javier Leon Serrano <javier.leon@unican.es>
Subject: RV: ONCSIS-20-0232RR Initial Quality Check

This email was sent to you by someone outside the University.
You should only click on links or attachments if you are certain that the email is genuine and the content is safe.

Dear author
The journal Oncogenesis is asking for the agreement of all the authors to the author list of the resubmitted version of the manuscript
As you can see in their instructions below, I would need that you send me a simple email confirming that you agree with the changes. I will combine your response emails in a document and submit it to the journal. I attach the final version and I copy the list of authors below

Judit Liaño-Pons1,5, M. Carmen Lafita-Navarro1,6, Lorena García-Gaipo1, Carlota Colomer2, Javier Rodríguez3, Alex von Kriegsheim3, Peter Hurlin4, Fabiana Ourique1,7, M. Dolores Delgado1, Anna Bigas2, M. Lluis Espinosa2 and Javier Leon1

Best regards
Javier

Prof. Javier León
Instituto de Biomedicina y Biotecnología de Cantabria (IBBTEC)
C/ Albert Einstein 22, 39011 Santander

Universidad de Cantabria-CSIC-Sodercan
Tel 34 942 201952; Mov 34 637 858 317

Leon Serrano, Javier

De: Hurlin, Peter <phurlin@shrinenet.org>
Enviado el: lunes, 30 de noviembre de 2020 23:09
Para: Leon Serrano, Javier
Asunto: Re: Authors agreement

Javier, Sorry for the delay, I was away for Thanksgiving holiday here.

I agree to the changes made to the manuscript.

Peter

Peter Hurlin PhD
Principal Investigator
Shriners Hospitals for Children
Associate Professor
Department of Orthopaedics and Rehabilitation
Department of Cell, Developmental and Cancer Biology
Oregon Health & Science University
Portland, OR
97239

From: Leon Serrano, Javier <javier.leon@unican.es>
Sent: Monday, November 30, 2020 8:31:28 AM
To: Hurlin, Peter
Subject: [Ext] RV: Authors agreement

De: Leon Serrano, Javier
Enviado el: viernes, 27 de noviembre de 2020 9:35
Para: phurlin@shrinenet.org
Asunto: Authors agreement

Dear Peter,
Please remember to send me this agreement. A sentence saying that you agree with eh list of authors of the resubmission will be enough. Thanks a lot
Best
Javier

De: Leon Serrano, Javier
Enviado el: jueves, 26 de noviembre de 2020 12:21
Para: judit.liano.pons@ki.se; MariadelCarmen.LafitaNavarro@UTSouthwestern.edu; Garcia Gaipo, Lorena <lorena.garcia@unican.es>; carlotacolomer@gmail.com; Rodriguez Martinez, Javier <javier.rodriguez@unican.es>; Alex.VonKriegsheim@igmm.ed.ac.uk; phurlin@shrinenet.org; fabiana.ourique@posgrad.ufsc.br; delgadmd@unican.es; abigas@imim.es; lespinosa@imim.es; leonj@unican.es
Asunto: RV: ONCSIS-20-0232RR Initial Quality Check

Dear author

Leon Serrano, Javier

De: fabiana.ourique@posgrad.ufsc.br
Enviado el: jueves, 26 de noviembre de 2020 12:44
Para: Leon Serrano, Javier
Asunto: Re: RV: ONCSIS-20-0232RR Initial Quality Check
Datos adjuntos: image001.jpg

Dear Prof Javier

I agree with the changes of the resubmitted version of the manuscript.

Best regards

Fabiana

Em 26.11.2020 08:21, Leon Serrano, Javier escreveu:

> Dear author
>
> The journal Oncogenesis is asking for the agreement of all the authors to the author list of the resubmitted version of the manuscript
>
> As you can see in their instructions below, I would need that you send me a simple email confirming that you agree with the changes. I will combine your response emails in a document and submit it to the journal. I attach the final version and I copy the list of authors below
>
> Judit Liaño-Pons1,5, M. Carmen Lafita-Navarro1,6, Lorena García-Gaipo1, Carlota Colomer2, Javier Rodríguez3, Alex von Kriegsheim3, Peter Hurlin4, Fabiana Ourique1,7, M. Dolores Delgado1, Anna Bigas2, M. Lluis Espinosa2 and Javier Leon1
>
> Best regards
>
> Javier

Prof. Javier León
Instituto de Biomedicina y Biotecnología de Cantabria (IBBTEC)
C/ Albert Einstein 22, 39011 Santander

Universidad de Cantabria-CSIC-Sodercan

Tel 34 942 201952; Mov 34 637 858 317

Leon Serrano, Javier

De: Delgado Villar, Maria Dolores
Enviado el: viernes, 27 de noviembre de 2020 12:09
Para: Leon Serrano, Javier
Asunto: RE: Agreeement on author list

Dear Javier,
I agree with the changes in the author list of the resubmitted version of the manuscript to Oncogenesis: Judit Liaño-Pons1,5, M. Carmen Lafita-Navarro1,6, Lorena García-Gaipo1, Carlota Colomer2, Javier Rodríguez3, Alex von Kriegsheim3, Peter Hurlin4, Fabiana Ourique1,7, M. Dolores Delgado1, Anna Bigas2, M. Lluis Espinosa2 and Javier Leon1
Best regards,
Dolores

Prof. M. Dolores Delgado
Instituto de Biomedicina y Biotecnología de Cantabria (IBBTEC)
C/ Albert Einstein 22. 39011 Santander.
UNIVERSIDAD DE CANTABRIA-CSIC-SODERCAN

Tel. + 34 942 201 998
Email: delgadmd@unican.es

Antes de imprimir este mensaje, asegúrate de que es necesario. Proteger el medio ambiente está en tus manos.

De: Leon Serrano, Javier <javier.leon@unican.es>
Enviado el: viernes, 27 de noviembre de 2020 12:01
Para: Delgado Villar, Maria Dolores <maria.delgado@unican.es>; Garcia Gaipo, Lorena <lorena.garcia@unican.es>; judit.liano.pons@ki.se; Maria del Carmen Lafita Navarro <MariadelCarmen.LafitaNavarro@UTSouthwestern.edu>
Asunto: Agreeement on author list

Hola colegas
A continuación os adjunto la lista de los coautores que aún faltan por enviarme el email con el visto bueno de la lista de autores de Oncogenesis:

- Peter Hurlin
- Dolores Delgado

☺
jav

De: Leon Serrano, Javier
Enviado el: jueves, 26 de noviembre de 2020 12:21
Para: judit.liano.pons@ki.se; MariadelCarmen.LafitaNavarro@UTSouthwestern.edu; Garcia Gaipo, Lorena <lorena.garcia@unican.es>; carlotacolomer@gmail.com; Rodriguez Martinez, Javier <javier.rodriguez@unican.es>; Alex.VonKriegsheim@igmm.ed.ac.uk; phurlin@shrinenet.org; fabiana.ourique@posgrad.ufsc.br; delgadmd@unican.es; abigas@imim.es; lespinosa@imim.es; leonj@unican.es
Asunto: RV: ONCSIS-20-0232RR Initial Quality Check

Dear author
The journal Oncogenesis is asking for the agreement of all the authors to the author list of the resubmitted version of the manuscript
As you can see in their instructions below, I would need that you send me a simple email confirming that you agree with the changes. I will combine your response emails in a document and submit it to the journal. I attach the final version and I copy the list of authors below

Judit Liaño-Pons1,5, M. Carmen Lafita-Navarro1,6, Lorena García-Gaipo1, Carlota Colomer2, Javier Rodríguez3, Alex von Kriegsheim3, Peter Hurlin4, Fabiana Ourique1,7, M. Dolores Delgado1, Anna Bigas2, M. Lluis Espinosa2 and Javier Leon1

Leon Serrano, Javier

De: Anna Bigas <abigas@imim.es>
Enviado el: jueves, 26 de noviembre de 2020 19:32
Para: Leon Serrano, Javier
Asunto: Re: ONCSIS-20-0232RR Initial Quality Check

Dear Javier, thanks so much for letting me participate in this study. We agree with all the changes made.
Sincerely, Anna Bigas.

El jue, 26 nov 2020 a las 12:21, Leon Serrano, Javier (<javier.leon@unican.es>) escribió:

> Dear author
>
> The journal Oncogenesis is asking for the agreement of all the authors to the author list of the resubmitted version of the manuscript
>
> As you can see in their instructions below, I would need that you send me a simple email confirming that you agree with the changes. I will combine your response emails in a document and submit it to the journal. I attach the final version and I copy the list of authors below
>
> Judit Liaño-Pons1,5, M. Carmen Lafita-Navarro1,6, Lorena García-Gaipo1, Carlota Colomer2, Javier Rodríguez3, Alex von Kriegsheim3, Peter Hurlin4, Fabiana Ourique1,7, M. Dolores Delgado1, Anna Bigas2, M. Lluis Espinosa2 and Javier Leon1
>
> Best regards
>
> Javier

Prof. Javier León
Instituto de Biomedicina y Biotecnología de Cantabria (IBBTEC)
C/ Albert Einstein 22, 39011 Santander

Universidad de Cantabria-CSIC-Sodercan

Tel 34 942 201952; Mov 34 637 858 317

De: oncogenesis@nature.com <oncogenesis@nature.com>
Enviado el: jueves, 26 de noviembre de 2020 11:30
Para: Leon Serrano, Javier <javier.leon@unican.es>
Asunto: ONCSIS-20-0232RR Initial Quality Check

Dear Professor León,

In checking in your manuscript submitted to Oncogenesis it has come to our attention that the following

Leon Serrano, Javier

De: Espinosa Blay, Lluis <lespinosa@imim.es>
Enviado el: jueves, 26 de noviembre de 2020 13:36
Para: Leon Serrano, Javier
Asunto: RE: ONCSIS-20-0232RR Initial Quality Check
Datos adjuntos: image003.jpg

Dear Javier,

I agree with all changes included in the last version of manuscript ONCSIS-20-0232RR.

Best wishes,

Lluís Espinosa, PhD
Cancer Research Program
IMIM-Hospital del Mar
PRBB
Dr. Aiguader, 88
08003, Barcelona. Spain
Tel:+34 93 3160589
e-mail: lespinosa@imim.es

____

De: Leon Serrano, Javier [javier.leon@unican.es] Enviat el: dijous, 26 / novembre / 2020 12:21 Per a: judit.liano.pons@ki.se; MariadelCarmen.LafitaNavarro@UTSouthwestern.edu; Garcia Gaipo, Lorena; carlotacolomer@gmail.com; Rodriguez Martinez, Javier; Alex.VonKriegsheim@igmm.ed.ac.uk; phurlin@shrinenet.org; fabiana.ourique@posgrad.ufsc.br; Delgado Villar, Maria Dolores; Bigas Salvans, Anna; Espinosa Blay, Lluis; Leon Serrano, Javier
Tema: RV: ONCSIS-20-0232RR Initial Quality Check

Dear author
The journal Oncogenesis is asking for the agreement of all the authors to the author list of the resubmitted version of the manuscript As you can see in their instructions below, I would need that you send me a simple email confirming that you agree with the changes. I will combine your response emails in a document and submit it to the journal. I attach the final version and I copy the list of authors below

Judit Liaño-Pons1,5, M. Carmen Lafita-Navarro1,6, Lorena García-Gaipo1, Carlota Colomer2, Javier Rodríguez3, Alex von Kriegsheim3, Peter Hurlin4, Fabiana Ourique1,7, M. Dolores Delgado1, Anna Bigas2, M. Lluis Espinosa2 and Javier Leon1

Best regards
Javier

[logoweb]Prof. Javier León
Instituto de Biomedicina y Biotecnología de Cantabria (IBBTEC) C/ Albert Einstein 22, 39011 Santander Universidad de Cantabria-CSIC-Sodercan Tel 34 942 201952; Mov 34 637 858 317

De: oncogenesis@nature.com <oncogenesis@nature.com> Enviado el: jueves, 26 de noviembre de 2020 11:30
Para: Leon Serrano, Javier <javier.leon@unican.es>
Asunto: ONCSIS-20-0232RR Initial Quality Check

Dear Professor León,
